# Supplementary material for: “What are you carrying?” Experiences of mothers with preterm babies in low-resource setting neonatal intensive care unit: a qualitative study
Source: BMJ Open. 2021 Sep 14;11(9):e043989. doi: 10.1136/bmjopen-2020-043989 (PMC8442070; doi:10.1136/bmjopen-2020-043989)
Supplement: Supplementary data [file bmjopen-2020-043989supp001.pdf]

Appendix: In depth Interview Guide

Understanding the Lived Experiences of Parents Caring For Preterm Babies in MNRH Neonatal Special Care Unit.

Thank you for agreeing to be interviewed. The kind of interview that we are going to have requires that I use an audio recorder so that I can later write our conversation without missing out important information. I am therefore requesting that you permit me to use the audio recorder. The information will be deleted once I am finished with the writing and no one else will have access to what you say or your recorded voice.

(*Turn on audio recorder*)

Thanks for agreeing to be interviewed for this research project. I'm hopeful that the information you and the other participants share with me will help provide information that provides insight into the lived experiences of parents caring for preterm babies at MNRH.

In order to understand your experience as I need to know about your strategies, and how having a preterm baby has affected your life. I have a set of questions to guide our conversation. I want to understand your experiences, and thoughts about caring for preterm baby

Time Started________________________________ Time Ended________________

What is your Age ___________________________

Religion ____

Occupation____

Marital status____

Level of education____

Obstetrical History:

Number of:

Pregnancies ___________________________

Live births: ______________________________

Still births: _______________________________

Miscarriages______________________________

Number of living children___________________

1. Tell me what you have gone through from the time there was indication that you would not be carrying your baby to full term
    a. In terms of what happened
    b. In terms of what went on in your mind eg when you first saw the baby
    c. In terms of the care and support
2. What do you think could have been the cause for the preterm birth?
3. What do you think could be the cause for having a preterm delivery?
4. Tell me about your thoughts regarding having your baby cared for in the Mulago National Referral Hospital?
5. What challenges have you faced in taking care of a preterm baby? (Prompts: taking care of others in your home, work schedules, financial challenges in this new role of looking after a preterm baby)
6. What support have you sought in the care of your preterm baby?
7. How can help to support mothers with preterm babies?

INTERVIEWER _________________________DATE INTERVIEW _______
