# Supplementary material for: “What are you carrying?” Experiences of mothers with preterm babies in low-resource setting neonatal intensive care unit: a qualitative study
Source: BMJ Open. 2021 Sep 14;11(9):e043989. doi: 10.1136/bmjopen-2020-043989 (PMC8442070; doi:10.1136/bmjopen-2020-043989)
Supplement: Author's manuscript [file bmjopen-2020-043989.draft_revisions.pdf]

BMJ Open is committed to open peer review. As part of this commitment we make the peer review history of every article we publish publicly available.

When an article is published we post the peer reviewers' comments and the authors' responses online. We also post the versions of the paper that were used during peer review. These are the versions that the peer review comments apply to.

The versions of the paper that follow are the versions that were submitted during the peer review process. They are not the versions of record or the final published versions. They should not be cited or distributed as the published version of this manuscript.

BMJ Open is an open access journal and the full, final, typeset and author-corrected version of record of the manuscript is available on our site with no access controls, subscription charges or pay-per-view fees (http://bmjopen.bmj.com).

If you have any questions on BMJ Open's open peer review process please email info.bmjopen@bmj.com

'What are you carrying?' Lived experiences of mothers with preterm babies in low resource setting neonatal intensive care unit - a qualitative study

Journal:	*BMJ Open*
Manuscript ID	bmjopen-2020-043989
Article Type:	Original research
Date Submitted by the Author:	21-Aug-2020

Complete List of Authors:	Namusoke, Fatuma; Makerere University, Obstetrics and Gynaecology<br>Sekikubo, Musa; Makere University/Mulago National Referral Hospital, Obstetrics and Gynecology<br>Namiiro, Flavia; Makerere University, Paediatrics and Child Health<br>Nakigudde, Janet ; Makerere University, Psychiatry

Keywords:	Maternal medicine < OBSTETRICS, Neonatal intensive & critical care < INTENSIVE & CRITICAL CARE, PRIMARY CARE

SCHOLARONE™
Manuscripts

I, the Submitting Author has the right to grant and does grant on behalf of all authors of the Work (as defined in the below author licence), an exclusive licence and/or a non-exclusive licence for contributions from authors who are: i) UK Crown employees; ii) where BMJ has agreed a CC-BY licence shall apply, and/or iii) in accordance with the terms applicable for US Federal Government officers or employees acting as part of their official duties; on a worldwide, perpetual, irrevocable, royalty-free basis to BMJ Publishing Group Ltd ("BMJ") its licensees and where the relevant Journal is co-owned by BMJ to the co-owners of the Journal, to publish the Work in this journal and any other BMJ products and to exploit all rights, as set out in our licence.

The Submitting Author accepts and understands that any supply made under these terms is made by BMJ to the Submitting Author unless you are acting as an employee on behalf of your employer or a postgraduate student of an affiliated institution which is paying any applicable article publishing charge ("APC") for Open Access articles. Where the Submitting Author wishes to make the Work available on an Open Access basis (and intends to pay the relevant APC), the terms of reuse of such Open Access shall be governed by a Creative Commons licence – details of these licences and which Creative Commons licence will apply to this Work are set out in our licence referred to above.

Other than as permitted in any relevant BMJ Author's Self Archiving Policies, I confirm this Work has not been accepted for publication elsewhere, is not being considered for publication elsewhere and does not duplicate material already published. I confirm all authors consent to publication of this Work and authorise the granting of this licence.

For peer review only

'What are you carrying?': `Lived experiences of mothers with preterm babies in low resource setting neonatal intensive care unit - a qualitative study

Authors: [1,2] *Fatuma Namusoke PhD, [1,2]Musa Sekikubo PhD, [4,5]Flavia Namiiro MMED, [1,3]Janet Nakigudde PhD

Affiliation:

[1]School of Medicine Makerere University

[2]Department of Obstetrics and Gynaecology

[3]Department of Psychiatry

[4]Mulago National Referral hospital

[5]Department of Pediatrics and Child Health

Emails:

Fatuma Namusoke : namusokefk@yahoo.co.uk

Musa Sekikubo:msekikubo@yahoo.com

Flavia Namiiro :bnflaviah@gmail.com

For peer review only

Janet Nakigudde: janetnakigudde@gmail.com

*Corresponding author:

Fatuma Namusoke,

Makerere University,

College of Health Sciences,

Department of Obstetrics and Gynaecology,

P.O. Box 7072, Kampala Uganda.

Tel: 0752613577 Email: namusokefk@yahoo.co.uk

Journal: BMJ open

Word count: 5,394

Abstract:

Introduction

Babies born preterm tend to suffer complications that can be reduced through admission and care in a neonatal intensive care unit (NICU). This separation of the mother from their vulnerable newborn though desired may impair bonding and lead to psychological distress in the mother.

Objective

This study explored the lived experiences of mothers with preterm babies admitted to a neonatal intensive care unit in a low resource setting.

For peer review only

Study design

The study used qualitative methods with both phenomenological in-depth interviews and focus group discussions to further highlight the lived experiences.

Study setting

Data was collected from a public hospital which works as a district and national referral hospital located in the capital.

Participants

Fifty one mothers with preterm babies in the neonatal intensive care unit were purposively sampled and recruited after informed consent. Data was analyzed using manual thematic analysis.

Results

Six thematic areas of concern for mothers of preterm babies in the NICU were; constant worry and uncertainty about the survival of their babies, baby feeding challenges, worries of discharge, communication gaps between mothers and nurses, financial and community acceptability of a preterm infant.

Conclusions and recommendations

The nurses and community are crucial for the support of the mothers with preterm babies in the neonatal intensive care unit in a low resource setting. Mothers should be supported with knowledge of preterm delivery during the antenatal clinics.

Key words: Preterm birth, Mothers, Lived experiences, low resource setting

Article summary

Strength and limitations of this study

- The study used qualitative methods with in-depth interviews and focus group discussions to document the lived experiences of mothers with preterm babies.
- Data collection on individual experiences of mothers with preterm babies was done using in-depth interviews and Focus group discussion to elicit data on a broad overview of the problem.

For peer review only

- To ease sharing of experiences by participants, all researchers involved in data collection were female and one of them was a clinician in the same hospital.
- Further probing where necessary was done to clarify the understanding of the lived experiences
- Data collection was done in the hospital settings which may have compromised the quality of data since participants were still receiving care.

Introduction

Globally, the under-five mortality rates decreased from 69.4 to 34.4 per 100,000 live births between 2000 and 2016 [1]. More than 40% of the deaths in the under-fives occur in the neonatal period [2]. Preterm birth is a major cause of morbidity and mortality in the neonatal period [3]. Preterm birth is a risk factor for half of all neonatal deaths [4] and is the second most significant cause of death in children below five years after pneumonia [3]. Globally, 60% of all preterm births occur in Sub-Saharan Africa and Asia [5] . Preterm babies who survive in the neonatal period may suffer short- and long-term complications.

Preterm babies suffer morbid conditions like difficulty in feeding, poor body temperature regulation, respiratory distress syndrome and intracranial hemorrhage [6]. Those that survive complications associated with preterm birth are prone to long term complications like developmental delays, blindness and cerebral palsy [3, 4]. Immediate, short- and long-term complications of preterm birth are significantly reduced under the care of qualified and dedicated personnel in Neonatal Intensive Care Units (NICUs) around the world [7].

The neonatal intensive care unit is a scary experience with noises, alarms going off, babies on respirators, need for expression of breast milk and sometimes the babies die while receiving care. Preterm birth comes too soon when the family is not psychologically prepared. Stress and anxiety are common in mothers caring for babies in the NICU [8]. The condition of the neonate and gestational age at delivery are the

For peer review only

major contributing factor to stress of parents. In Sub-Saharan Africa, 12·3% (9·5–15·8) of all deliveries are preterm [5].

Breast milk is the best feed recommended for the preterm babies [9] and yet expressing it is not culturally acceptable because it is erroneously associated with neonatal death. The high patient to nurse ratio, overcrowding of the wards and limited bed capacity of the neonates in the low resource settings presents a unique challenge to the parents. The admission of a preterm baby to the neonatal intensive care unit soon after delivery leads to separation from the mother.

This separation presents a dual challenge to the mother coping with her puerperium, nursing a preterm and worrying about the uncertain outcomes of her newborn. Studies have highlighted different experiences by mothers delivering preterm babies ranging from anxiety through distress to depression in the parents [10-12]. This has led to development of techniques like family centered care which is keen on the experiences of the parents as they take care of the preterm baby [13, 14]. However, it is still not clear what parents of the preterm babies in a low resource setting are facing given the multiple challenges.

This study explored the lived experiences of mothers caring for preterm babies in a neonatal intensive care unit in a low resource setting. This is important in tailoring the interventions to support these mothers.

Methods

This was a qualitative study with both phenomenological in-depth interviews and focus group discussions to stimulate and open up new ideas on experiences of mothers with preterm. We explored the experiences of the women that had had preterm babies admitted to the NICU. The in-depth interviews explored the experiences and focus group discussions generated a deeper understanding of the phenomena of interest. Considering that the topic of preterm babies might be sensitive but not confidential, the information from the groups helped to validate the information from the in-depth interviews. Information was collected at two time points for the women. The women

For peer review only

whose babies were still admitted in the neonatal intensive care unit were subjected to in-depth interviews. The women who were organized into focus groups were women whose babies were stable and they were bringing them back for review and immunization.

Study setting: Mulago National Referral hospital is located in Uganda's capital city and is one of the busiest maternity and neonatal centers in the country. The neonatal intensive care unit admits preterm infants and newborns with complications from the hospital as well as referrals from clinics and hospitals around the country. The NICU at Mulago National Referral hospital admits 7-15 neonates in 24 hours. It has a total bed capacity of 49 cots but usually admits up to three times its capacity that averages 400 admissions per month with more than half of them due to prematurity [15]. During the time of the study, Mulago National Referral Hospital had its department of Obstetrics and Gynecology and NICU transferred to Kawempe division due to the ongoing renovation of the main hospital. This further compromised the space available to cater for the same number of neonates. Mulago national referral hospital is a public hospital that offers free services. The patients /attendants are sometimes asked to buy drugs and supplies used in the newborn care. Most of the medicines used on the preterm are not available on the hospital essential drug list. In addition the patients cater for their sustenance during the hospital stay.

Study population and Sample:

Women who had delivered live babies at less than 37 weeks of gestation and admitted in the neonatal intensive care unit were purposively sampled. Mothers with a live preterm baby (assessed using modified Ballard score on admission to NICU), whose infant had been admitted to NICU for at least five days were included in the study after informed consent. Mothers whose neonates were very ill or had a congenital abnormality and/or were unable to communicate in the local language; Luganda or English were excluded from in-depth interview (IDI). The Inclusion criteria for the focus group discussions were mothers who had their preterm neonate admitted to the NICU, discharged and had then come back for review in the neonatal follow-up clinic. Mothers

For peer review only

who had participated in the in-depth interviews and those unable to communicate in English or the local language in the area were excluded from focus group discussion (FGD).

Study procedure

The data collection involved interviews of the parents in private consultation (in depth interviews) using a guide which is attached as appendix. Data on demographic characteristics of the participants, namely age, numbers of living children and educational level was collected.

Data was in addition collected using FGDs of 10-12 participants who had delivered preterm babies admitted in the NICU. These were discharged and scheduled for review by the pediatric team. Participants were purposively sampled and recruited after informed consent.

The IDIs and FDGs were face to face within the hospital premises. All the interviews were conducted by one investigator and one note taker with training in qualitative research. Another investigator (FN) took part in the focus group discussions. FN is an obstetrician gynaecologist in the hospital. All the researchers involved in data collection were female and fluent in both English and Luganda; one of the major languages spoken in Uganda. The interview lasted for 30 to 40 minutes. The interview was conducted guided by a semi-structured interview guide. All the interviews were transcribed and audio taped.

The mothers of preterm neonates admitted to the NICU were recruited after informed written consent. The male partners were not present at the time of the interview. Data collection started with collecting information on the demographics of the study participants. The parents were asked to describe their experiences when they learnt that they would be delivering a preterm baby. Then they were asked to reflect on what they thought could have led to delivery of the preterm infant. They were then asked to reflect on the care of their preterm baby at the national referral hospital NICU. Then, the challenges encountered were elicited from the participants. In order to further

For peer review only

understand the phenomenon any further questions that arose during the interview were pursued for clarification. Data collection was continued until no new data was generated.

We used a qualitative research design with a modified grounded theoretical basis in collecting and managing the data. Sixteen in-depth interviews and four focus group discussions were conducted with women that had delivered a preterm baby from one day to six months. All participants that were approached to participate in this study agreed to participate. However one woman in a focus group discussion had a neonate that appeared not to be breathing well. This was noted by the obstetrician who was seated in the focus group discussion. This woman was taken out before the group commenced so that her baby could immediately be attended to by the pediatrician on duty. A total of 51 research participants were involved in this study. Interviews were audio taped, transcribed and analyzed using manual thematic analysis.

Data analysis

Data were analyzed using manual thematic analysis. Two of the authors (JN and FN) familiarized themselves with the data from the transcripts after the data cleaning. Initial preliminary codes were then generated. On further familiarization with the data, patterns in the codes were generated and a further exploration of this data provided us with primary and secondary codes and then finally themes were generated and defined. Each major theme that was generated was illustrated using the quotations from the women. The process of the data analysis was not sequential but iterative because on numerous occasions, the two authors would engross themselves in the data, back and forth until they agreed on the final themes. Six themes generated were constant worry and uncertainty about the survival of their babies, baby feeding challenges, worries of discharge, communication gaps between mothers and nurses, financial and community acceptability of a preterm infant.

Ethical considerations

The study was approved by Makerere University School of Medicine Research and Ethics Committee (REF 2018-092) and the Uganda National Council of Science and

For peer review only

Technology (HS 2457). Administrative clearance for the study was obtained from the director of the hospital. The patients were approached through the nurse in charge who acted as the gate keeper. She informed them about the study. Participants were included after informed written consent. Participants were informed that participation was voluntary, and that although the study would not benefit them directly, the findings of the study could impact on future care of preterm and their mothers. Arrangements were made with the hospital counselor for any of the participants who would get emotional distress as a result of the interview. None of the participants required counseling following the interview.

Patient/public involvement

The participants were not involved with the conception, design or conduct of the study. The public was involved in vetting and final approval of the study protocol. The public has permanent representation on the ethical committees that approved the study. The results of the study will be disseminated to the participants through the media.

Results

Demographics

Fifty-one mothers were included in both in depth interviews and focus group discussions, 34/51 (66.67%) were married, more than 70% of them delivered the baby between 23 to 29 weeks of gestation. Details of other demographic characteristics of the study participants are shown in Table 1.

Table 1: Demographic characteristics of mothers with preterm babies

**N = 51**	**Frequency**	**Percentage**
**Mother's age in completed years**		
**17-25**	24	47
**26-35**	24	47

For peer review only

**36-45**	3	6
**Marital Status**		
**Married**	34	67
**Single**	17	33
**Education in Years**		
**<7 years**	17	34
**8-12 years**	20	39
**>13**	14	27
**Number of pregnancies**		
**1-3**	33	65
**4-8**	18	35
**Number of live births**		
**1-3**	41	80
**4-8**	11	20
**Gestation of delivery of current baby in weeks**		
**23-29**	36	71
**>30**	15	29

228

229 ## Themes

230 Six themes on women's experiences of having a preterm baby were developed after
231 data analysis. The themes include uncertainty about the survival of a preterm baby,
232 feeding challenges of the baby, worries about care of the baby after discharge,
233 communication gap between mothers and nurses and the perceived insensitivity of the
234 nurses, community acceptability and disdain for preterm babies and financial challenges
235 of having a preterm baby.

For peer review only

Theme 1: Uncertainty about the survival of a preterm baby

A number of women with preterm babies at the national referral hospital were worried and uncertain whether their newly born babies survive. One mother who had delivered twins illustrated this fear in an In-depth interview quotation below:

> *"I worry more about taking care of my twins, I do not know if they will live or not though they are under good care by the health workers"*(In-depth 12)

One mother describes how the changing weight of her preterm baby was constantly a concern and how she would always run back to hospital because the baby's weight had decreased:

> *"I was very worried! I even feared the baby and I was asking myself of how long are we going to be in this type of care or if the child will survive anyway! I was discharged after a week! So you can imagine how scared I was. But whenever the weight would go down I had to run back to hospital".* (FGD 2; respondent 6)

The weight of a preterm baby was constantly a worry to the mothers and they would constantly worry that due to the baby's tiny size, the baby would not survive. This is illustrated in the quotation below:

> *"My baby was too tiny that I would even fear to hold it. I would only be told by the other mothers that when you give birth to a premature baby he is taken to the Special care unit and kept there, so I would always worry about my baby's size and ask my self if he will live."* (FGD 4 R 5)

A mother reported that there was too much death in the neonatal special care unit and that every time her baby was moved to a new crib it was because the baby that had been in that space had died and her baby would be replacing the dead one.

> *"Every time they moved my baby to a different bed, it was replacing one that had passed on and so I kept asking myself, whether my baby would die next?"* (FGD 2; respondent 1)

For peer review only

Mothers were anxious about taking care of their babies; they had feeding challenges and reported that they could go through all the other challenges if only they were sure that their babies would survive. There was reported agony of not knowing that one's baby was going to survive. This is illustrated in the following quotation:

> "Going back home is one of our least worries if we only knew that we can access help and assistance. But what is most worrisome is the impending looming death of one's baby!" (FGD 4 R5)

Theme 2: Feeding challenges of a preterm baby

Feeding of preterm babies had a number of challenges for the mothers; many of the neonates had to be fed through a tiny feeding tube. These are inserted because most preterm babies have difficulty in feeding. Inserting the feeding tube when a mother is discharged was a challenge. This challenge was coupled with maintaining good hygiene and ensuring the baby does not vomit the milk. This is pointed out in the following quotation by a mother:

> *"Sometimes they discharge children with feeding tubes and that tube requires so much care and good hygiene but also the babies keep vomiting when fed through these tubes. We are not taught well on how to give the milk while at home."* (FGD 2; Respondent1)

On the same issue of feeding another mother with a lot of dismay pointed out that:

> *"My baby pulled out the tube and the nurse warned me that it would go into the lungs. However she did not help me put it back!."* (FGD 2; respondent 5)

Further, some mothers had complaints with keeping the inserted feeding tube in place. This required the mother to express the milk but also to learn to feed the baby using the tubes. This could be a challenge to the mothers as is indicated below in this quotation:

For peer review only

"Using a tube to feed the baby is not easy! When the feeding tube comes out you have to run to the hospital and yet the tube easily comes out." (FGD 2 respondent 1).

Theme 3: Worries about care of the baby after discharge

Mothers felt that the clinicians supported them as long as they were still in hospital. It was very distressing for them to be told that they would be discharged because then they would have to accept the whole burden of taking care of their preterm babies' special needs amidst the insecurities of not knowing what to do in case of any eventuality with the baby. The women felt that they would not have access to immediate help if their preterm babies' wellbeing deteriorated while at home.

"I worry about how I will be with my baby after we have been discharged since I have no idea on how to take care of a premature baby." (In-depth 12).

Two mothers from in-depth interviews expressed their lack of knowledge of taking care of preterm babies on their own. This is expressed in the two quotes below.

"I am not confident enough when it comes to taking care of premature babies since I have no knowledge. (In-depth 13)

"*Our babies need a lot of attention and time, yet sometimes you are discharged, you are alone at home and you are even afraid of the baby*." (FGD 4 R3)

Theme 4: Communication gap between mothers and nurses and the perceived insensitivity of the nurses

There seemed to be a communication gap between the nurses and the mothers. Some mothers complained that when they would want to talk to the nurses regarding the care of their babies, the nurses would ignore them as is illustrated in the quotes below:

"It is always challenging that the health workers tend to ignore us when we call for their assistance." (In-depth 6)

For peer review only

Poor communication between the clinicians and the mothers was further illustrated on occasions when mothers would be given prescriptions for medicines to buy for their babies. The mothers would assume that since they had purchased the medications, these medications would be administered to the babies. Instances would occur when the mothers would later find out that the medicines that had been requested of them were not administered to babies and no explanations would be given to the mothers. This is pointed out in the quotation:

> *"I was told to go buy the medication from a pharmacy outside the hospital which I did, but every time I asked the doctor to tell me about the progress of the baby, they wouldn't tell me anything not even about administering the medication that they requested me to buy."* (FGD 2; respondent 6)

Further a lack of clear communication was indicated when mothers indicated that they were lost for how to go about a baby who developed other medical issues. One mother kept on wondering if she should bring back her baby for care because the baby now had developed flu as is described below:

> *"We are given review dates to come back, then my baby developed flu, but it was not yet time to bring the baby back for review! So I kept wondering if I had to comeback earlier or wait for the review date, I had been given."*(FGD 4 R2)

Theme 5: Community acceptability and disdain for preterm babies

Mothers with preterm babies are always told to protect their babies from catching infections and they are told not to give their babies to people when they come to visit. When a visitor comes to see the baby and is not given the baby to see because the mothers have been advised against this, the purpose for which he has come is not fulfilled and this may lead to a confrontation. This stresses the mothers as is shown in the quotation:

> *"We were told not to always expose our babies because they can easily catch infections, so when I went home, I had to hide my baby. So many people felt*

For peer review only

offended and they would always complain that I do not want to show them my baby." (FGD 4 R4)

The community where the mothers are coming from appeared to not give as much value to preterm babies as the mothers themselves and this was agonizing and insensitive to the mothers. One mother reported that people would ask her what she was carrying even when they well knew that she was carrying a baby:

"Back home when most people would see my baby, they would even ask me" 'what is that you are carrying?' and yet they very well know you are carrying a baby but they say it just to make you feel bad. But you have no option except to continue tolerating people and putting your baby under the sun to work on the yellowing skin" (FGD 4 R4)

Theme 6: Financial Challenges of having a preterm baby

The government hospitals offer free services to mothers and their babies. In cases where the medicines and supplies are not available, the patients are asked to buy from a private pharmacy. All patients are required to cater for their sustenance during the hospital stay. When a baby is born, there are expected expenditures that many new parents will prepare for. However, it is hard and expensive to envisage preparing for a preterm baby who has uniquely expensive needs in terms of time, care and emotionally. Many mothers were worried on how they would manage to financially meet the obligations of the baby and also of the other obligations that they were taking care of before they delivered the preterm baby. This is what they had to say:

"I worry about how I will take care of my child financially! I need to have money for diapers, tubes and clothes and yet I have had to put my business to a halt so that I can give time to and take care of my baby!." (In-depth 2)

Another mother with other financial responsibilities in the home and a need to go back to work said that:

" *I was also worried on how I would now take care of the people at home and the finances to take care of my baby is very hard since my husband is a Taxi driver.*

For peer review only

Sometimes you need to buy medication, diapers. I also worry that I will not be in position to work immediately since I have to stay home and take care of my baby". (In-depth 12)

What you pay for when you go to hospital with a preterm baby is way above what a mother would pay for if she had a term baby. Most of the medicine used in the care of the preterm baby is not on the essential drug list and usually costly. Taking care of the preterm requires prolonged stay in the hospital since the mothers are required to stay in hospital all the time as long the baby is admitted. This leads to interference in the income generating activities of the family. The financial challenges faced by these mothers are expressed in the quote below;

" It has also been a challenge in terms of finances due to the high demands while in hospital because the medications are expensive!". (In-depth 8)

For peer review only

Discussion

The study explored the lived experiences of women who had delivered preterm babies in a low income setting. The mothers were concerned about survival of their newborn babies, coping with the feeding schedules of the preterm and poor community attitude towards preterm babies. Mothers in Sweden were pre-occupied with guilt and blame in addition to balancing the role of mother and spouse.[10] Delivery of a preterm baby is a traumatic experience to the parents [16].

Uncertainty about the survival of a preterm baby/ Worrying about the preterm babies' survival

The mothers of the preterm infants were worried about their survival Mothers having their preterm baby in a neonatal intensive care unit and looking at how the other babies were dying left them in suspense about survival of their preterm infants. This is consistent with previous studies which indicated that mothers of hospitalized infants are worried about whether their infant will survive [17, 18]. Looking at the changes in the weight of the baby with time compounded their anxiety as most of the preterm neonates lose weight before gaining weight. The risk of death of the preterm babies in the low- and middle-income countries continues into the post neonatal period [19]. The survival is less in the developing countries where facilities for prevention of mortality in the antenatal period and early neonatal period are better

Feeding challenges of a preterm baby

Our findings indicate that mothers were generally challenged about feeding the preterm. The preterm babies have an immature digestive which leads to many feeding problems. The PTB frequently cannot coordinate suckling, swallow and breathing, tend to have gastric residues due to incomplete emptying, gastrooesophageal reflux and abdominal distension. In order to overcome these problems the preterm babies receive gavage feeding where a feeding tube is placed through the nose or mouth to the stomach. This allows for easy monitoring of the feeding of the preterm infant. The babies are given feeding tubes which are perceived by the parents to hurt their vulnerable infant. This finding is consistent with other studies done in developed countries where the mothers

For peer review only

also expressed challenges in feeding the preterm [20]. Feeding a preterm baby is a challenge since these have not had neuromuscular development which is essential in feeding.. This is traumatic to the mother as she feels that her baby may be hurt.

Worries about discharge

Mothers were constantly worried about discharge of the baby. They were not sure whether they were ready to look after their preterm without the supervision of the health worker. Previous studies have showed that parents of preterm babies get a lot of anxiety and worry when it comes to discharge of the baby [8, 16]. There are no standardized criteria for discharge of preterm babies and developing this has been suggested as one of the ways to decrease anxiety of the mother [21]. The mothers knowing criteria for discharge would reduce the anxiety once they understand that it has been tested.

Perceived insensitivity of the Nurses in the neonatal unit

Some mothers of the preterm babies felt that the nurses caring for their babies were insensitive about the challenges they were facing while taking care of their babies. Crowded NICUs constrain the time a single nurse/ clinician will spend with every baby and mother which leads to prioritizing the very ill. To overcome this problem group sessions regarding care of newborns are given and not for the individual mothers. Using this approach, there may not be an opportunity to assess Individual needs and gaps for each mother. Where mothers are not able to get the support from health workers in care of their preterm they resorted to peer support [18]. Babies in the NICU are cared for but mothers may not have good emotional support and information which would affect their perception of care [22].

Communication gap between mothers and nurses

Participants in this study felt that the health workers were not communicating well to them not giving them time to ask any questions regarding the condition of the baby and treatment given. Previous studies have alluded to obtaining similar research findings where women caring for preterm babies in the NICU valued being listened to and having a formal communication with the health professionals [11, 23]. Lack of

For peer review only

communication and indifference of the health workers leave the mothers with a preterm baby helpless since these are in charge of their babies. The communication problem in this group is due to the high patient load with less than optimal staffing levels in the public health setting [24]. Increase workload of nurses working in the neonatal intensive care unit has been associated with missing nursing care previously [25]. Giving the right information to the mothers with preterm labour by the health workers helps to empower them on decision making [26].

Community acceptability of preterm babies

The community where the mothers are coming from appeared to not give as much value to preterm babies or the challenges the mothers were going through. Mothers felt that the community and family members did not give them support when they delivered the preterm baby. Our findings were unique when it came to community acceptability of a preterm baby. There was no study where the mothers of preterm were worried about what the other members of the community thought about their baby. Uganda is one of the countries in Africa with a high fertility rate and a high regard to the number of children [27]. Delivering a child is taken as an event for the community who feel that they have a right to know see the baby all the time. Most studies looking at experience of mothers with preterm babies are done in developed countries where close family members are involved in child care. Where all concerned have to see and care for the baby, a preterm baby ceases to contribute to the oneness of the community because the baby will be kept away for health reasons and people may not even see the baby until it is safe to have him out.

Financial Challenges of having a preterm baby

Mothers caring for preterm infants in the NICU are faced with financial difficulties and poverty as was also found [where?][28] .The mothers are faced with many financial obligations and have to invest a lot of time while taking care of their preterm infant as has been described [18].

For peer review only

Limitations

The participants were from a busy neonatal intensive care unit in a national referral hospital. The results from this study may not be generalized to all mothers of preterm in low resource setting. This study however presents the experiences of mothers in resource limited setting seeking care in a public health care facility. The mothers of preterm babies were still receiving care in the facility which could have biased the results. Data was collected while the baby is NICU to avoid recall bias.

Conclusions

Making a journey as a woman with a preterm baby was anxiety provoking because of the uncertainties for survival, challenges in care, communication problems with the health workers and the harsh treatment of the community following discharge.

Recommendation:

We recommend that mothers attending the antenatal clinic are educated about preterm birth and the how this can be handles. Mothers attending to babies in the NICU be organized in support groups as they go through the stressful time.

Funding statement:

This work was supported by Bill and Melinda Gates Foundation through the 'Preterm Birth initiative' project to the University of California San Francisco grant number OPP1107312.

Competing interests statement

Authors declare no competing interests.

Author's contribution

All the authors contributed to conception of the study. FN and JN collected and analysed the data. FN, MS, FN AND JN have critically reviewed the manuscript for intellectual content. All authors approved the final version of the manuscript and are accountable for all aspects of the work presented here.

For peer review only

For peer review only - http://bmjopen.bmj.com/site/about/guidelines.xhtml

Acknowledgements

We would like to acknowledge the research participants who provided information on which this writing is based. We are very grateful to the partners and family that supported the mothers during the hospital stay. We would also like to acknowledge Professor Derek Pomeroy of derekp@cantab.net for proof reading the article.

Data sharing statement

Data will be available on request.

References

1. Collaborators, G.B.D.M., *Global, regional, and national under-5 mortality, adult mortality, age-specific mortality, and life expectancy, 1970-2016: a systematic analysis for the Global Burden of Disease Study 2016.* Lancet, 2017. **390**(10100): p. 1084-1150.
2. Liu, L., et al., *Global, regional, and national causes of child mortality: an updated systematic analysis for 2010 with time trends since 2000.* Lancet, 2012. **379**(9832): p. 2151-61.
3. Blencowe, H., et al., *Born too soon: the global epidemiology of 15 million preterm births.* Reprod Health, 2013. **10 Suppl 1**: p. S2.
4. Lawn, J.E., et al., *3.6 million neonatal deaths--what is progressing and what is not?* Semin Perinatol, 2010. **34**(6): p. 371-86.
5. Hannah Blencowe , S.C., Mikkel Z Oestergaard, Doris Chou, Ann-Beth Moller, Rajesh Narwal, Alma Adler, Claudia Vera Garcia, Sarah Rohde, Lale Say, Joy E Lawn, *National, regional, and worldwide estimates of preterm birth rates in the year 2010 with time trends since 1990 for selected countries: a systematic analysis and implications.* Lancet, 2012. **9**(379(9832):): p. 2162-72.
6. Platt, M.J., *Outcomes in preterm infants.* Public Health, 2014. **128**(5): p. 399-403.
7. Cats, B.P. and N. Beganovic, *Intensive care for sick neonates, the earlier the better: improved survival rate of IRDS children, referred by means of a mobile neonatal intensive care unit.* J Perinat Med, 1979. **7**(1): p. 19-22.
8. Saenz, P., et al., *Psychological stress of parents of preterm infants enrolled in an early discharge programme from the neonatal intensive care unit: a prospective randomised trial.* Arch Dis Child Fetal Neonatal Ed, 2009. **94**(2): p. F98-F104.
9. Ahrabi, A.F. and R.J. Schanler, *Human milk is the only milk for premies in the NICU!* Early Hum Dev, 2013. **89 Suppl 2**: p. S51-3.
10. Lundqvist, P., J. Weis, and B. Sivberg, *Parents' journey caring for a preterm infant until discharge from hospital-based neonatal home care-A challenging process to cope with.* J Clin Nurs, 2019. **28**(15-16): p. 2966-2978.
11. Sawyer, A., et al., *Parents' experiences and satisfaction with care during the birth of their very preterm baby: a qualitative study.* BJOG, 2013. **120**(5): p. 637-43.
12. Dadkhahtehrani, T., et al., *Experiences of Fathers with Inpatient Premature Neonates: Phenomenological Interpretative Analysis.* Iran J Nurs Midwifery Res, 2018. **23**(1): p. 71-78.
13. Zhang, R., et al., *Involvement of Parents in the Care of Preterm Infants: A Pilot Study Evaluating a Family-Centered Care Intervention in a Chinese Neonatal ICU.* Pediatr Crit Care Med, 2018. **19**(8): p. 741-747.

For peer review only

14. Ding, X., et al., *Effects of family-centred care interventions on preterm infants and parents in neonatal intensive care units: A systematic review and meta-analysis of randomised controlled trials.* Aust Crit Care, 2019. **32**(1): p. 63-75.
15. Abdallah, Y., et al., *Is facility based neonatal care in low resource setting keeping pace? A glance at Uganda's National Referral Hospital.* Afr Health Sci, 2016. **16**(2): p. 347-55.
16. O'Donovan, A. and E. Nixon, *"Weathering the storm:" Mothers' and fathers' experiences of parenting a preterm infant.* Infant Ment Health J, 2019. **40**(4): p. 573-587.
17. Docherty, S.L., M.S. Miles, and D. Holditch-Davis, *Worry about child health in mothers of hospitalized medically fragile infants.* Adv Neonatal Care, 2002. **2**(2): p. 84-92.
18. Lomotey, A.Y., et al., *Experiences of mothers with preterm babies at a Mother and Baby Unit of a tertiary hospital: A descriptive phenomenological study.* Nurs Open, 2020. **7**(1): p. 150-159.
19. Katz, J., et al., *Mortality risk in preterm and small-for-gestational-age infants in low-income and middle-income countries: a pooled country analysis.* Lancet, 2013. **382**(9890): p. 417-425.
20. Russell, G., et al., *Parents' views on care of their very premature babies in neonatal intensive care units: a qualitative study.* BMC Pediatr, 2014. **14**: p. 230.
21. Quinn, J.M., M. Sparks, and S.M. Gephart, *Discharge Criteria for the Late Preterm Infant: A Review of the Literature.* Adv Neonatal Care, 2017. **17**(5): p. 362-371.
22. De Rouck, S. and M. Leys, *Information needs of parents of children admitted to a neonatal intensive care unit: a review of the literature (1990-2008).* Patient Educ Couns, 2009. **76**(2): p. 159-73.
23. Ward, K., *Perceived needs of parents of critically ill infants in a neonatal intensive care unit (NICU).* Pediatr Nurs, 2001. **27**(3): p. 281-6.
24. Namaganda, G., et al., *Making the transition to workload-based staffing: using the Workload Indicators of Staffing Need method in Uganda.* Hum Resour Health, 2015. **13**: p. 89.
25. Tubbs-Cooley, H.L., et al., *Association of Nurse Workload With Missed Nursing Care in the Neonatal Intensive Care Unit.* JAMA Pediatr, 2019. **173**(1): p. 44-51.
26. Gaucher, N. and A. Payot, *From powerlessness to empowerment: Mothers expect more than information from the prenatal consultation for preterm labour.* Paediatr Child Health, 2011. **16**(10): p. 638-42.
27. Kabagenyi, A., et al., *Has Uganda experienced any stalled fertility transitions? Reflecting on the last four decades (1973-2011).* Fertil Res Pract, 2015. **1**(14): p. 1-10.
28. Gondwe, A., et al., *Perceptions and experiences of community members on caring for preterm newborns in rural Mangochi, Malawi: a qualitative study.* BMC Pregnancy Childbirth, 2014. **14**: p. 399.

For peer review only

Appendix: In depth Interview Guide

Understanding the Lived Experiences of Parents Caring For Preterm Babies in MNRH Neonatal Special Care Unit.

Thank you for agreeing to be interviewed. The kind of interview that we are going to have requires that I use an audio recorder so that I can later write our conversation without missing out important information. I am therefore requesting that you permit me to use the audio recorder. The information will be deleted once I am finished with the writing and no one else will have access to what you say or your recorded voice.

(*Turn on audio recorder*)

Thanks for agreeing to be interviewed for this research project. I'm hopeful that the information you and the other participants share with me will help provide information that provides insight into the lived experiences of parents caring for preterm babies at MNRH.

In order to understand your experience as I need to know about your strategies, and how having a preterm baby has affected your life. I have a set of questions to guide our conversation. I want to understand your experiences, and thoughts about caring for preterm baby

Time Started________________________________Time Ended________________

What is your Age ____________________________

Religion ____

Occupation____

Marital status____

Level of education____

Obstetrical History:

Number of:

Pregnancies ____________________________

Live births: ______________________________

Still births: _______________________________

For peer review only

Miscarriages______________________________

Number of living children___________________

1. Tell me what you have gone through from the time there was indication that you would not be carrying your baby to full term
    a. In terms of what happened
    b. In terms of what went on in your mind eg when you first saw the baby
    c. In terms of the care and support
2. What do you think could have been the cause for the preterm birth?
3. What do you think could be the cause for having a preterm delivery?
4. Tell me about your thoughts regarding having your baby cared for in the Mulago National Referral Hospital?
5. What challenges have you faced in taking care of a preterm baby? (Prompts: taking care of others in your home, work schedules, financial challenges in this new role of looking after a preterm baby)
6. What support have you sought in the care of your preterm baby?
7. How can help to support mothers with preterm babies?

INTERVIEWER __________________________DATE INTERVIEW _______

For peer review only

SUPPLEMENT 1: COREQ 32-ITEM CHECKLIST

Tong A, Sainsbury P, Craig J. (2007) Consolidated criteria for reporting qualitative research (COREQ): a 32-item checklist for interviews and focus groups. International Journal for Quality in Healthcare: 19:349 – 357

No. Item	Guide questions/description	Reported on Page #
Domain 1: Research team and reflexivity		
1. Inter viewer/facilitator	Which author/s conducted the interview?	7
2. Credentials	What were the researcher's credentials?	1,7
3. Occupation	What was their occupation at the time of the study?	7
4. Gender	Was the researcher male or female?	7
5. Experience and training	What experience or training did the researcher have?	7
6. Relationship with participants established	Was a relationship established prior to study commencement?	8
7. Participant knowledge of the interviewer	What did the participants know about the researcher?	8
8. Interviewer characteristics	What characteristics were reported about the inter viewer/facilitator?	8
Domain 2: study design		
9. Methodological orientation and Theory	What methodological orientation was stated to underpin the study?	6
10. Sampling	How were participants selected?	8
11. Method of approach	How were participants approached?	8
12. Sample size	How many participants were in the study?	8
13. Non-participation	How many people refused to participate or dropped out? Reasons?	8
14. Setting of data collection	Where was the data collected?	6
15. Presence of non-participants	Was anyone else present besides the participants and researchers?	7
16. Description of sample	What are the important characteristics of the sample?	5,6 and Table 1
17. Interview guide	Were questions, prompts, guides provided by the authors?	7
18. Repeat interviews	Were repeat interviews carried out?	9
19. Audio/visual recording	Did the research use audio or visual recording to collect the data?	7
20. Field notes	Were field notes made during and/or	7

For peer review only

	after the inter view?	
21. Duration	What was the duration of the interviews	8
22. Data saturation	Was data saturation discussed?	8
23. Transcripts returned	Were transcripts returned to participants for comment and/or correction?	No
Domain 3: analysis and findings		
24. Number of data coders	How many data coders coded the data?	2
25. Description of the coding tree	Did authors provide a description of the coding tree?	no
26. Derivation of themes	Were themes identified in advance or derived from the data?	8
27. Software	What software, if applicable, was used to manage the data?	No
28. Participant checking	Did participants provide feedback on the findings?	No
29. Quotations presented	Were participant quotations presented to illustrate the themes/findings? Was each quotation identified?	10-16
30. Data and findings consistent	Was there consistency between the data presented and the findings?	10-16
31. Clarity of major themes	Were major themes clearly presented in the findings?	10-16
32. Clarity of minor themes	Is there a description of diverse cases or discussion of minor themes?	10-16

For peer review only

BMJ Open

‘What are you carrying?’: `Experiences of mothers with preterm babies in low resource setting neonatal intensive care unit - a qualitative study

Journal:	*BMJ Open*
Manuscript ID	bmjopen-2020-043989.R1
Article Type:	Original research
Date Submitted by the Author:	25-Apr-2021
Complete List of Authors:	Namusoke, Fatuma; Makerere University, Obstetrics and Gynaecology<br>Sekikubo, Musa; Makere University/Mulago National Referral Hospital, Obstetrics and Gynecology<br>Namiiro, Flavia; Makerere University, Paediatrics and Child Health<br>Nakigudde, Janet ; Makerere University, Psychiatry
**Primary Subject Heading**:	Health services research
Secondary Subject Heading:	Obstetrics and gynaecology, Paediatrics
Keywords:	Maternal medicine < OBSTETRICS, Neonatal intensive & critical care < INTENSIVE & CRITICAL CARE, PRIMARY CARE

SCHOLARONE™
Manuscripts

I, the Submitting Author has the right to grant and does grant on behalf of all authors of the Work (as defined in the below author licence), an exclusive licence and/or a non-exclusive licence for contributions from authors who are: i) UK Crown employees; ii) where BMJ has agreed a CC-BY licence shall apply, and/or iii) in accordance with the terms applicable for US Federal Government officers or employees acting as part of their official duties; on a worldwide, perpetual, irrevocable, royalty-free basis to BMJ Publishing Group Ltd ("BMJ") its licensees and where the relevant Journal is co-owned by BMJ to the co-owners of the Journal, to publish the Work in this journal and any other BMJ products and to exploit all rights, as set out in our licence.

The Submitting Author accepts and understands that any supply made under these terms is made by BMJ to the Submitting Author unless you are acting as an employee on behalf of your employer or a postgraduate student of an affiliated institution which is paying any applicable article publishing charge ("APC") for Open Access articles. Where the Submitting Author wishes to make the Work available on an Open Access basis (and intends to pay the relevant APC), the terms of reuse of such Open Access shall be governed by a Creative Commons licence – details of these licences and which Creative Commons licence will apply to this Work are set out in our licence referred to above.

Other than as permitted in any relevant BMJ Author's Self Archiving Policies, I confirm this Work has not been accepted for publication elsewhere, is not being considered for publication elsewhere and does not duplicate material already published. I confirm all authors consent to publication of this Work and authorise the granting of this licence.

For peer review only

‘*What are you carrying*?’: `Experiences of mothers with preterm babies in low resource setting neonatal intensive care unit - a qualitative study

Authors: [1,2] *Fatuma Namusoke [1,2]Musa Sekikubo , [4,5]Flavia Namiiro , [1,3]Janet Nakigudde

Affiliation:

[1]School of Medicine Makerere University

[2]Department of Obstetrics and Gynaecology

[3]Department of Psychiatry

[4]Mulago National Referral hospital

[5]Department of Pediatrics and Child Health

Emails:

Fatuma Namusoke: namusokefk@yahoo.co.uk

Musa Sekikubo: msekikubo@yahoo.com

Flavia Namiiro: bnflaviah@gmail.com

Janet Nakigudde: janetnakigudde@gmail.com

For peer review only

*Corresponding author:

Fatuma Namusoke,

Makerere University,

College of Health Sciences,

Department of Obstetrics and Gynaecology,

P.O. Box 7072, Kampala Uganda.

Tel: 0752613577 Email: namusokefk@yahoo.co.uk

Journal: BMJ open

Word count: 5,394

For peer review only

Abstract:

Introduction

Babies born preterm often have challenges in feeding, temperature control, breathing difficulty and are prone to infection during the neonatal period. These usually necessitate admission to the neonatal intensive care unit (NICU). The admission to NICU disrupts the mother baby bonding.

Objective

This study explored the lived experiences of mothers with preterm babies admitted to a neonatal intensive care unit in a low resource setting.

Study design

This was a qualitative study where 16 participants took part in the in-depth interviews and 35 in the focus group discussions. We included mothers who delivered and were caring for preterm babies at the Neonatal Intensive care unit at Mulago National Referral Hospital.

Study setting

Data were collected from a public hospital, which works as a district and national referral hospital located in the capital of Uganda.

Participants

Fifty-one mothers with preterm babies in the neonatal intensive care unit were sampled and recruited after informed consent. Data was analyzed using manual thematic analysis.

Results

Six themes in experiences mothers of preterm babies in NICU were constant worry and uncertainty about the survival of their babies, baby feeding challenges, worries of discharge, communication gaps between mothers and nurses, community acceptability and disdain for preterm babies and financial challenges.

For peer review only

Conclusions and recommendations

Mothers of preterm babies admitted to NICU in a low resource setting still need a lot of support other than the medical care given to their babies. Support groups in the hospital and community are recommended to help in dealing with these challenges.

Key words: Preterm birth, mothers, experiences, low resource setting

Article summary

Strength and limitations of this study

- The study used qualitative methods with in-depth interviews and focus group discussions to document the experiences of mothers with preterm babies.
- To ease sharing of experiences by participants, all researchers involved in data collection were female and one of them was a clinician in the same hospital.
- Further probing where necessary was done to clarify the understanding of the experiences
- Data collection was done in the hospital settings, which may have compromised the quality of data since participants were still receiving care.

Introduction

Globally, the under-five mortality rates decreased significantly from 69.4 to 34.4 per 100,000 live births between 2000 and 2016 [1] but only modestly in developing countries. More than 40% of the deaths in the under-fives occur in the neonatal period [2]. Preterm birth is a major cause of morbidity and mortality in the neonatal period [3] and accounts for half of all neonatal deaths [4]. Babies born preterm and survive the neonatal period suffer short- and long-term complications.

In Uganda, preterm delivery accounts for 14% live births and 28% of the neonatal deaths [5]. Despite decreases in under-five mortality, neonatal mortality rate did not change between 2006 and 2016 [6]. More than 50% of all low birth weight babies in

For peer review only

Uganda are preterm. In 2011, more than three quarters of low birth weight babies in Uganda died in the neonatal period [7].

Additionally, preterm babies have poor body temperature regulation, respiratory distress syndrome and intracranial hemorrhage that may further compromise their survival [8]. Admission of preterm babies is therefore essential to avert short- and medium-term complications in this patient category. However, due to limited medical supplies required in the critical care of preterm babies in developing countries, mortality and long-term morbidity are disproportionately high.

Breast milk is the best food recommended for the preterm babies [9] and pumping is required as preterm babies have difficulty lurching on the breast; it is a costly practice in developing countries [10]. Mothers need support to correctly and hygienically collect breast milk for their preterm babies but this is hampered by the high patient to nurse ratio.

Mulago National Referral Hospital is the main government maternity hospital serving the metropolitan area of Kampala and receives referrals from district and regional hospitals. The population of Kampala metropolitan area is estimated to be 3,138,000 people of whom (percent of women of reproductive age). The neonatal intensive care unit (NICU) has limited infrastructure and skilled health care providers to deal with the high number of high risk small and sick newborns as reported in health care services giving care to the sick/small newborn [11]. Other NICU facilities (mostly level 2) available in Kampala are paid for and include one private not for profit hospital and two private hospitals at a limited scale. The admission of a preterm baby to the neonatal intensive care unit soon after delivery leads to separation from the mother.

This separation presents a dual challenge to the mother coping with her puerperium, nursing a preterm and worrying about the uncertain outcomes of her newborn. It is still not clear what mothers of the preterm babies in a low resource setting are facing given the multiple challenges, which is the subject of this study.

Methods

For peer review only

A total of 52 mothers were included, 16 in the I in-depth interviews (IDI) and four focus group discussions (FGD). The study was conducted in a period of two months. The sequence of conducting IDI followed by FGDs was to use the FGDs to further explore the phenomena identified in the IDIs.

Study setting: Mulago National Referral hospital is located in Uganda's capital city and is one of the busiest maternity and neonatal centers in the country. The neonatal intensive care unit admits preterm infants and newborns with complications from the hospital as well as referrals from clinics and hospitals around the country. In 2020, the NICU had 4,572 admissions with (2345/4572) 51% preterm born within the facility (hospital records). It has a total bed capacity of 49 cots but usually admits up to three times its capacity that averages 400 admissions per month with more than half of them due to prematurity [12]. The team caring preterm babies includes six pediatricians, 19 nurses and 4 nursing assistants. The nurses working at the NICU are trained and dedicated to the unit. The nurses work in a 12hr shift, with more allocations in the day compared to night duty. The average nurse patient ratio in the NICU is 1:35. Babies admitted to the NICU are assessed for gestational age using modified Ballard score. During the time of the study, Mulago National Referral Hospital had its department of Obstetrics and Gynecology and NICU transferred to Kawempe division due to the ongoing renovation of the main hospital. The relocation compromised the space available to cater for the same number of neonates. Apart from treatment given, Family Nurture Interventions (FNI) in the NICU are done to prevent the adverse effects of separating mothers from their preterm. The FNI offered during the time of study was skin to skin care during the time of feeding. Kangaroo mother care services were not offered because of space limitations at Kawempe. The mothers/attendants are sometimes asked to buy drugs and supplies like surfactant, antibiotics and feeding tubes used in the newborn cater for their sustenance during the hospital stay.

Study population and Sample:

Women who had delivered live babies at less than 37 weeks of gestation and admitted in the neonatal intensive care unit were purposively sampled and recruited after

For peer review only

informed consent. The IDI were conducted on mothers of preterm whose babies were still in the NICU at the time of interview. Mothers whose neonates were very ill or had a congenital abnormality and/or were unable to communicate in the local language (Luganda) or English were excluded from in-depth interview (IDI). The women who were organized into focus groups had their preterm baby/babies in NICU, discharged and brought baby for review during the study period. All mothers included in the FGD had babies below three completed months of age. Mothers who participated in the in-depth interviews were excluded from focus group discussion.

Study procedure

The depth interviews and focus group discussions were face to face conducted in a private consultation within the hospital premises. Eligible mothers were interviewed between the intervals of feeding the babies. The FGDs consisted of 10-12 participants who were identified when they brought their babies for review or treatment by the pediatric team. The patients were approached through the nurse in charge who acted as the gatekeeper who informed them about the study. All participants approached agreed to participate in the study. However, one woman in a focus group discussion had a neonate that appeared not to be breathing well. This was noted by the obstetrician who was seated in the focus group discussion. This woman was taken out before the group commenced so that her baby could immediately be attended to by the pediatrician on duty. Data collection was done by two investigators (JN and FN) and one note taker with training in qualitative research. FN is an obstetrician gynaecologist in the hospital. All the researchers involved in data collection were female and fluent in both English and Luganda. The interview lasted for 30 to 40 minutes. The interview was conducted guided by a semi-structured interview guide which is attached as an appendix. All the interviews were audio taped and transcribed.

The male partners were not present at the time of the interview. Data collection started with collecting information on the demographics of the study participants. Mothers were asked to describe their experiences when they learnt that they would be delivering a preterm baby. Then they were asked to reflect on what they thought could have led to

For peer review only

delivery of the preterm infant. They were then asked to reflect on the care of their preterm baby at the NICU and challenges encountered. In order to further understand the phenomenon any further questions that arose during the interview were pursued for clarification. Data collection was continued until no new data were generated. Data collection and management was done according to the COREQ guidelines for qualitative studies.

Data analysis

Data were analyzed using manual thematic analysis. Two of the authors (JN and FN) familiarized themselves with the data from the transcripts after the data cleaning and generated preliminary codes. On further familiarization with the data, patterns in the codes were generated and a further exploration of this data provided us with primary and secondary codes and then finally themes were generated and defined. Each major theme generated was illustrated using the quotations from the women. The process of the data analysis was not sequential but iterative because on numerous occasions, the two authors would engross themselves in the data, back and forth until they agreed on the final themes.

Ethical considerations

The study was approved by Makerere University School of Medicine Research and Ethics Committee (REF 2018-092) and the Uganda National Council of Science and Technology (HS 2457). Administrative clearance for the study was obtained from Mulago National Referral Hospital administration. Participants were included after informed written consent. Participants were informed that participation was voluntary, with no direct benefit to the participants or their neonates. The findings of the study could impact on future care of preterm babies and their mothers. Arrangements were made with the hospital counselor for any of the participants who would become emotionally distressed as a result of the interview. None of the participants required counseling following the interview. In Uganda individuals below the age of majority who are pregnant or have a child are considered as emancipated minors and may independently give consent to participate in research approved by institutional review committee [13].

For peer review only

Patient/public involvement

The participants were not involved with the conception, design or conduct of the study. The public was involved in vetting and final approval of the study protocol. The public has permanent representation on the ethical committees that approved the study. The results of the study will be disseminated to the participants through the media.

Results

Demographics

Fifty-one mothers were included in both in depth interviews and focus group discussions, 34/51 (66.67%) were married, more than 70% of them delivered the baby between 23 to 29 weeks of gestation. Details of other demographic characteristics of the study participants are as shown in Table 1.

Table 1: Demographic characteristics of mothers with preterm babies

N = 51	Frequency	Percentage
**Mother's age in completed years**		
**= or >18**	5	9.8
**19-25**	19	37.25
**26-35**	24	47.0
**36-45**	3	6.0
**Marital Status**		
**Married**	34	67.0
**Single**	17	33.0
**Education in Years**		
**<7 years**	17	34.0
**8-12 years**	20	39.0
**>13**	14	27.0
**Number of pregnancies**		

For peer review only

**1-3**	33	65.0
**4-8**	18	35.0
**Number of live births**		
**1-3**	41	80.0
**4-8**	11	20.0
**Gestation of delivery of current baby in weeks**		
**23-29**	36	71
**>30**	15	29

Themes

Six themes on women's experiences of having a preterm baby were developed after data analysis. The themes were uncertainty about the survival of a preterm baby, feeding challenges of the baby, worries about care of the baby after discharge, communication gap between mothers and nurses and the perceived insensitivity of the nurses, community acceptability and disdain for preterm babies and financial challenges of having a preterm baby.

Theme 1: Uncertainty about the survival of a preterm baby

A number of women with preterm babies at the national referral hospital were worried and uncertain whether their newly born babies would survive. One mother who had delivered twins illustrated this fear in an In-depth interview quotation below:

> *"I worry more about taking care of my twins, I do not know if they will live or not though they are under good care by the health workers"* (In-depth 12)

One mother describes how the changing weight of her preterm baby was constantly a concern and how she would always run back to hospital because the baby's weight had decreased:

> *"I was very worried! I even feared the baby and I was asking myself of how long are we going to be in this type of care or if the child will survive anyway! I was*

For peer review only

discharged after a week! So you can imagine how scared I was. But whenever the weight would go down I had to run back to hospital". (FGD 2; respondent 6)

The weight of a preterm baby was constantly a worry to the mothers and they would constantly worry that due to the baby's tiny size, the baby would not survive. This is illustrated in the quotation below:

"My baby was too tiny that I would even fear to hold it. I would only be told by the other mothers that when you give birth to a premature baby he is taken to the Special care unit and kept there, so I would always worry about my baby's size and ask my self if he will live." (FGD 4 R 5)

A mother reported that there was too much death in the neonatal special care unit and that every time her baby was moved to a new crib it was because the baby that had been in that space had died and her baby would be replacing the dead one.

"Every time they moved my baby to a different bed, it was replacing one that had passed on and so I kept asking myself, whether my baby would die next?" (FGD 2; respondent 1)

Mothers were anxious about taking care of their babies; they had feeding challenges and reported that they could go through all the other challenges if only they were sure that their babies would survive. There was reported agony of not knowing that one's baby was going to survive. This is illustrated in the following quotation:

"*Going back home is one of our least worries if we only knew that we can access help and assistance. But what is most worrisome is the impending looming death of one's baby!"* (FGD 4 R5)

Theme 2: Feeding challenges of a preterm baby

Feeding of preterm babies had a number of challenges for the mothers; many of the neonates had to be fed through a tiny feeding tube. These are inserted because most

For peer review only

preterm babies have difficulty in feeding. Inserting the feeding tube when a mother is discharged was a challenge. This challenge was coupled with maintaining good hygiene and ensuring the baby does not vomit the milk. This is pointed out in the following quotation by a mother:

> *"Sometimes they discharge children with feeding tubes and that tube requires so much care and good hygiene but also the babies keep vomiting when fed through these tubes. We are not taught well on how to give the milk while at home."* (FGD 2; Respondent1)

On the same issue of feeding another mother with a lot of dismay pointed out that:

> *"My baby pulled out the tube and the nurse warned me that it would go into the lungs. However she did not help me put it back!."* (FGD 2; respondent 5)

Further, some mothers had complaints with keeping the inserted feeding tube in place. This required the mother to express the milk but also to learn to feed the baby using the tubes. This could be a challenge to the mothers as is indicated below in this quotation:

> *"Using a tube to feed the baby is not easy! When the feeding tube comes out you have to run to the hospital and yet the tube easily comes out."* (FGD 2 respondent 1).

Theme 3: Worries about care of the baby after discharge

Mothers felt that the clinicians supported them as long as they were still in hospital. It was very distressing for them to be told that they would be discharged because then they would have to accept the whole burden of taking care of their preterm babies' special needs amidst the insecurities of not knowing what to do in case of any eventuality with the baby. The women felt that they would not have access to immediate help if their preterm babies' wellbeing deteriorated while at home.

For peer review only

"I worry about how I will be with my baby after we have been discharged since I have no idea on how to take care of a premature baby." (In-depth 12).

Two mothers from in-depth interviews expressed their lack of knowledge of taking care of preterm babies on their own. This is expressed in the two quotes below.

"I am not confident enough when it comes to taking care of premature babies since I have no knowledge. (In-depth 13)

"*Our babies need a lot of attention and time, yet sometimes you are discharged, you are alone at home and you are even afraid of the bab*y." (FGD 4 R3)

Theme 4: Communication gap between mothers and nurses and the perceived insensitivity of the nurses

There seemed to be a communication gap between the nurses and the mothers. Some mothers complained that when they would want to talk to the nurses regarding the care of their babies, the nurses would ignore them as is illustrated in the quotes below:

"It is always challenging that the health workers tend to ignore us when we call for their assistance." (In-depth 6)

Poor communication between the clinicians and the mothers was further illustrated on occasions when mothers would be given prescriptions for medicines to buy for their babies. The mothers would assume that since they had purchased the medications, these medications would be administered to the babies. Instances would occur when the mothers would later find out that the medicines that had been requested of them were not administered to babies and no explanations would be given to the mothers. This is pointed out in the quotation:

"I was told to go buy the medication from a pharmacy outside the hospital which I did, but every time I asked the doctor to tell me about the progress of the baby, they wouldn't tell me anything not even about administering the medication that they requested me to buy." (FGD 2; respondent 6)

For peer review only

Further a lack of clear communication was indicated when mothers said that they were lost for what to do for a baby who developed other medical issues. One mother kept on wondering if she should bring back her baby for care because the baby now had developed flu as described below:

> *"We are given review dates to come back, then my baby developed flu, but it was not yet time to bring the baby back for review! So I kept wondering if I had to comeback earlier or wait for the review date, I had been given."*(FGD 4 R2)

Theme 5: Community acceptability and disdain for preterm babies

Mothers with preterm babies are always told to protect their babies from catching infections and they are told not to give their babies to people when they come to visit. When a visitor comes to see the baby and do not give them the baby to see because the mothers have been advised against this, the purpose for which he has come is not fulfilled and this may lead to a confrontation. This stresses the mothers as is shown in the quotation:

> *"We were told not to always expose our babies because they can easily catch infections, so when I went home, I had to hide my baby. So many people felt offended and they would always complain that I do not want to show them my baby." (FGD 4 R4)*

The community where the mothers are coming from appeared not give as much value to preterm babies as the mothers themselves and this was agonizing and insensitive to the mothers. One mother reported that people would ask her what she was carrying even when they well knew that she was carrying a baby:

> *"Back home when most people would see my baby, they would even ask me" 'what is that you are carrying?' and yet they very well know you are carrying a baby but they say it just to make you feel bad. But you have no option except to continue tolerating people and putting your baby under the sun to work on the yellowing skin"* (FGD 4 R4)

For peer review only

Theme 6: Financial Challenges of having a preterm baby

The government hospitals offer free consultation services to mothers and their babies. In cases where the medicines and supplies are not available, the patients buy from a private pharmacy. All patients are required to cater for their sustenance during the hospital stay. When a baby is born, there are expected expenditures that many new mothers will prepare for. However, it is hard and expensive to envisage preparing for a preterm baby who has uniquely expensive needs in terms of time, care and emotionally. Many mothers were worried on how they would meet financial the obligations of the baby and those that they were taking care of before they delivered the preterm baby. This is what they had to say:

> *"I worry about how I will take care of my child financially! I need to have money for diapers, tubes and clothes and yet I have had to put my business to a halt so that I can give time to and take care of my baby!"* (In-depth 2)

Another mother with other financial responsibilities in the home and a need to go back to work said that:

> "*I was also worried on how I would now take care of the people at home and the finances to take care of my baby is very hard since my husband is a Taxi driver. Sometimes you need to buy medication, diapers. I also worry that I will not be in position to work immediately since I have to stay home and take care of my baby".* (In-depth 12)

What you pay for when you go to hospital with a preterm baby is way above what a mother would pay for if she had a term baby. Medicine like surfactant used in the care of the preterm baby is not on the Uganda essential drug list and usually costly. Taking care of the preterm requires prolonged stay in the hospital since the mothers are required to stay in hospital all the time of admission. This leads to interference in the income generating activities of the family. The financial challenges faced by these mothers are expressed in the quote below;

For peer review only

"It has also been a challenge in terms of finances due to the high demands while in hospital because the medications are expensive!" (In-depth 8)

For peer review only

Discussion

This study shows delivery of a preterm and consequent admission to the Neonatal Intensive Care Unit in a low resource setting is an emotional packed period with various challenges. The experience of the mothers during admission in the NICU shaped the way they looked at their surviving baby. These results are important in highlighting the unique challenges faced by mothers of preterm infants in NICU in a low resource setting.

The mothers were worried about the survival of their babies. Mothers having their preterm baby in a Neonatal Intensive Care Unit and looking at how the other babies were dying left them in suspense about survival of their preterm infants. This is consistent with previous studies, which indicated that mothers of hospitalized infants are worried about their survival [14, 15]. Looking at the changes in the weight of the baby with time compounded their anxiety as most of the preterm neonates lose weight before gaining. The risk of death of the preterm babies in the low- and middle-income continues into the post neonatal period [16]. The survival preterm neonate is less in the developing countries where facilities for prevention of mortality in the antenatal period and early neonatal period are largely lacking.

Our findings indicate that mothers were generally challenged about feeding the preterm. The preterm babies have an immature digestive system that leads to many feeding problems. The preterm babies frequently cannot coordinate suckling [17], swallowing and breathing; tend to have gastric residues due to incomplete emptying, gastroesophageal reflux and abdominal distension [18]. In order to overcome these problems, the preterm babies receive gavage feeding where a feeding tube is placed through the nose or mouth to the stomach. This allows for easy monitoring of the feeding of the preterm infant. The use of nasal gastric feeding tubes has been associated with higher rates of central apnea than oroenteric feeding [19]. The oroenteric feeding however has a challenge of securing the feeding tube in place. The

For peer review only

preterm babies are given feeding tubes which are perceived by the mothers to hurt their vulnerable infant.

Mothers were constantly worried about discharge of the baby. They were not sure whether they were ready to look after their preterm without the supervision of the health worker. Previous studies have shown that parents of preterm babies get a lot of anxiety and worry when it comes to discharge of the baby [20, 21]. There are no standardized criteria for discharge of preterm babies and developing this has been suggested as one of the ways to decrease anxiety of the mother [22]. The mothers knowing criteria for discharge would reduce the anxiety once they understand that it has been tested.

Some mothers of the preterm babies felt that the nurses caring for their babies were insensitive about the challenges they were facing while taking care of their babies. A study done in Ghana showed that where mothers are not able to get the support from health workers in care of their preterm they resorted to peer support [15]. Babies in the NICU are cared for but mothers may not have good emotional support and information, which would affect their perception of care [23]. Crowded NICUs are constrained by the time a single nurse/ clinician will spend with every baby and mother, which leads to prioritizing the very ill. To overcome this problem group sessions regarding care of newborns are given and not for the individual mothers. Using this approach, there may not be an opportunity to assess Individual needs and gaps for each mother

Participants in this study felt that the health workers were not communicating well to them nor giving them time to ask any questions regarding the condition of the baby and treatment given. Previous studies done in the developed countries have alluded to obtaining similar research findings where women caring for preterm babies in the NICU valued being listened to and having a formal communication with the health professionals [24, 25]. The communication problem in this setting may be due to the high patient load with less than optimal staffing levels in the public health setting [26]. Increased workload of nurses in the neonatal intensive care unit has been associated with missing nursing care previously [27]. Giving the right information to the mothers with preterm labor by the health workers helps to empower them in -decision-making

For peer review only

[28]. Lack of communication and indifference of the health workers leave the mothers with a preterm baby helpless since these are in charge of their babies.

The community where the mothers come from appeared not to give as much value to preterm babies or the challenges the mothers were going through. Mothers felt that the community and family members did not give them support when they delivered the preterm baby. Our findings were unique when it came to community acceptability of a preterm baby. There was no study where the mothers of a preterm were worried about what the other members of the community thought about their baby. Uganda is one of the countries in Africa with a high fertility rate and a high regard to the number of children [29]. Delivering a child is taken as an event for the community who feel that they have a right to see the baby all the time. Most studies looking at experience of mothers with preterm babies are done in developed countries where close family members are involved in childcare. Where all concerned have to see and care for the baby, a preterm baby ceases to contribute to the oneness of the community because the baby will be kept away for health reasons and people may not even see the baby until it is safe to have him/her out.

Mothers were faced with financial challenges as they looked after the preterm babies. Delivering a preterm infant has been found a present a high economic burden on the family through direct medical and non-medical costs as well as lost working days [30]. The mothers are faced with many financial obligations and have to invest a lot of time while taking care of their preterm infant as has been described [15]. These challenges are worse in financially constrained population in the low resource setting.

Limitations

The results from this study may not be generalized to all mothers of preterm babies in low resource settings. At the time of data collection, the facility had been shifted to another unit to allow for renovations in the hospital. The medical team and equipment used in care of the newborn were not affected by the shifting. This study however presents the experiences of mothers in resource-limited settings seeking care in a

For peer review only

public health care facility. The mothers of preterm babies were still receiving care in the facility, which could have biased the results.

Conclusions

Making a journey as a woman with a preterm baby was anxiety provoking because of the uncertainties for survival, challenges in care, communication problems with the health workers and the harsh treatment of the community following discharge.

Recommendation:

We recommend that mothers attending the antenatal clinic are educated about preterm birth and how this can be handled. We recommend that issues regarding financial preparation including saving for expenses that come at time of delivery should be included in the antenatal health talks. Financial empowerment of the women in reproductive age and educating the girl child in these settings is highly recommended as a strategy to avert the challenges. Mothers attending to babies in the NICU could be organized in support groups as they go through the stressful time. Family Nature interventions aiming at supporting mothers and their preterm infants are highly recommended as a solution to some of the challenges. A pilot study in the low resource setting is recommended before rolling it out owing to the diversity of challenges faced by mothers of preterm babies. We recommend increased funding for the health sector in an effort to decrease the patient nurse/ doctor ratio.

Funding statement:

The Bill and Melinda Gates Foundation supported this work through the ‘Preterm Birth initiative’ project to the University of California San Francisco grant number OPP1107312.

Competing interests statement

Authors declare no competing interests.

For peer review only

Author's contribution

All the authors contributed to conception of the study. FN and JN collected and analyzed the data. FN, MS, FN AND JN have critically reviewed the manuscript for intellectual content. All authors approved the final version of the manuscript and are accountable for all aspects of the work presented here.

Acknowledgements

We would like to acknowledge the research participants who provided information on which this writing is based. We are very grateful to the partners and family that supported the mothers during the hospital stay. We would also like to acknowledge Professor Derek Pomeroy for proof reading the article.

Data sharing statement

Data will be available on request.

Ethics statement

Prior to initiating data collection, the study was approved by Makerere University School of Medicine Research and Ethics Committee (REF 2018-092) and the Uganda National Council of Science and Technology (HS 2457). All study participants provided informed consent and those below the age of majority were considered as emancipated minors.

References

1. Collaborators, G.B.D.M., *Global, regional, and national under-5 mortality, adult mortality, age-specific mortality, and life expectancy, 1970-2016: a systematic analysis for the Global Burden of Disease Study 2016.* Lancet, 2017. **390**(10100): p. 1084-1150.
2. Liu, L., et al., *Global, regional, and national causes of child mortality: an updated systematic analysis for 2010 with time trends since 2000.* Lancet, 2012. **379**(9832): p. 2151-61.
3. Blencowe, H., et al., *Born too soon: the global epidemiology of 15 million preterm births.* Reprod Health, 2013. **10 Suppl 1**: p. S2.
4. Lawn, J.E., et al., *3.6 million neonatal deaths--what is progressing and what is not?* Semin Perinatol, 2010. **34**(6): p. 371-86.
5. W MCEE - World Health Organization, *WHO methods and data sources for child causes of death 2000–2015.* 2016.
6. USAID, *Trends and Determinants of Neonatal Mortality in Uganda: Further analysis of the Demographic and Health Surveys* 2019.
7. Arunda, M.O., A. Agardh, and B.O. Asamoah, *Survival of low birthweight neonates in Uganda: analysis of progress between 1995 and 2011.* BMC Pregnancy Childbirth, 2018. **18**(1): p. 189.
8. Platt, M.J., *Outcomes in preterm infants.* Public Health, 2014. **128**(5): p. 399-403.

For peer review only

9. Ahrabi, A.F. and R.J. Schanler, *Human milk is the only milk for premies in the NICU!* Early Hum Dev, 2013. **89 Suppl 2**: p. S51-3.
10. Felice, J.P., et al., *"Breastfeeding" but not at the breast: Mothers' descriptions of providing pumped human milk to their infants via other containers and caregivers.* Matern Child Nutr, 2017. **13**(3).
11. Narayanan, I., et al., *Facility readiness in low and middle-income countries to address care of high risk/ small and sick newborns.* Matern Health Neonatol Perinatol, 2019. **5**: p. 10.
12. Abdallah, Y., et al., *Is facility based neonatal care in low resource setting keeping pace? A glance at Uganda's National Referral Hospital.* Afr Health Sci, 2016. **16**(2): p. 347-55.
13. Technology, U.N.C.f.S.a., *NATIONAL GUIDELINES FOR RESEARCH INVOLVING HUMANS AS RESEARCH PARTICIPANTS.* http://research.ciu.ac.ug/files/National_Guidelines_for_Research_Involving_Human_Participants.pdf Accessed 24/2/21, 2007.
14. Docherty, S.L., M.S. Miles, and D. Holditch-Davis, *Worry about child health in mothers of hospitalized medically fragile infants.* Adv Neonatal Care, 2002. **2**(2): p. 84-92.
15. Lomotey, A.Y., et al., *Experiences of mothers with preterm babies at a Mother and Baby Unit of a tertiary hospital: A descriptive phenomenological study.* Nurs Open, 2020. **7**(1): p. 150-159.
16. Katz, J., et al., *Mortality risk in preterm and small-for-gestational-age infants in low-income and middle-income countries: a pooled country analysis.* Lancet, 2013. **382**(9890): p. 417-425.
17. DeMauro, S.B., et al., *Postdischarge feeding patterns in early- and late-preterm infants.* Clin Pediatr (Phila), 2011. **50**(10): p. 957-62.
18. Ewer, A.K., et al., *Gastric emptying and gastro-oesophageal reflux in preterm infants.* Arch Dis Child Fetal Neonatal Ed, 1996. **75**(2): p. F117-21.
19. van Someren, V., et al., *An investigation into the benefits of resiting nasoenteric feeding tubes.* Pediatrics, 1984. **74**(3): p. 379-83.
20. O'Donovan, A. and E. Nixon, *"Weathering the storm:" Mothers' and fathers' experiences of parenting a preterm infant.* Infant Ment Health J, 2019. **40**(4): p. 573-587.
21. Saenz, P., et al., *Psychological stress of parents of preterm infants enrolled in an early discharge programme from the neonatal intensive care unit: a prospective randomised trial.* Arch Dis Child Fetal Neonatal Ed, 2009. **94**(2): p. F98-F104.
22. Quinn, J.M., M. Sparks, and S.M. Gephart, *Discharge Criteria for the Late Preterm Infant: A Review of the Literature.* Adv Neonatal Care, 2017. **17**(5): p. 362-371.
23. De Rouck, S. and M. Leys, *Information needs of parents of children admitted to a neonatal intensive care unit: a review of the literature (1990-2008).* Patient Educ Couns, 2009. **76**(2): p. 159-73.
24. Sawyer, A., et al., *Parents' experiences and satisfaction with care during the birth of their very preterm baby: a qualitative study.* BJOG, 2013. **120**(5): p. 637-43.
25. Ward, K., *Perceived needs of parents of critically ill infants in a neonatal intensive care unit (NICU).* Pediatr Nurs, 2001. **27**(3): p. 281-6.
26. Namaganda, G., et al., *Making the transition to workload-based staffing: using the Workload Indicators of Staffing Need method in Uganda.* Hum Resour Health, 2015. **13**: p. 89.
27. Tubbs-Cooley, H.L., et al., *Association of Nurse Workload With Missed Nursing Care in the Neonatal Intensive Care Unit.* JAMA Pediatr, 2019. **173**(1): p. 44-51.
28. Gaucher, N. and A. Payot, *From powerlessness to empowerment: Mothers expect more than information from the prenatal consultation for preterm labour.* Paediatr Child Health, 2011. **16**(10): p. 638-42.

29. Kabagenyi, A., et al., *Has Uganda experienced any stalled fertility transitions? Reflecting on the last four decades (1973-2011).* Fertil Res Pract, 2015. **1**(14): p. 1-10.

30. Hodek, J.M., J.M. von der Schulenburg, and T. Mittendorf, *Measuring economic consequences of preterm birth - Methodological recommendations for the evaluation of personal burden on children and their caregivers.* Health Econ Rev, 2011. **1**(1): p. 6.

For peer review only

Appendix: In depth Interview Guide

Understanding the Lived Experiences of Parents Caring For Preterm Babies in MNRH Neonatal Special Care Unit.

Thank you for agreeing to be interviewed. The kind of interview that we are going to have requires that I use an audio recorder so that I can later write our conversation without missing out important information. I am therefore requesting that you permit me to use the audio recorder. The information will be deleted once I am finished with the writing and no one else will have access to what you say or your recorded voice.

(*Turn on audio recorder*)

Thanks for agreeing to be interviewed for this research project. I'm hopeful that the information you and the other participants share with me will help provide information that provides insight into the lived experiences of parents caring for preterm babies at MNRH.

In order to understand your experience as I need to know about your strategies, and how having a preterm baby has affected your life. I have a set of questions to guide our conversation. I want to understand your experiences, and thoughts about caring for preterm baby

Time Started________________________________Time Ended________________

What is your Age ____________________________

Religion ____

Occupation____

Marital status____

Level of education____

Obstetrical History:

Number of:

Pregnancies ____________________________

Live births: ______________________________

Still births: _______________________________

For peer review only

Miscarriages______________________________

Number of living children___________________

1. Tell me what you have gone through from the time there was indication that you would not be carrying your baby to full term
    a. In terms of what happened
    b. In terms of what went on in your mind eg when you first saw the baby
    c. In terms of the care and support
2. What do you think could have been the cause for the preterm birth?
3. What do you think could be the cause for having a preterm delivery?
4. Tell me about your thoughts regarding having your baby cared for in the Mulago National Referral Hospital?
5. What challenges have you faced in taking care of a preterm baby? (Prompts: taking care of others in your home, work schedules, financial challenges in this new role of looking after a preterm baby)
6. What support have you sought in the care of your preterm baby?
7. How can help to support mothers with preterm babies?

INTERVIEWER __________________________DATE INTERVIEW _______

For peer review only

SUPPLEMENT 1: COREQ 32-ITEM CHECKLIST

Tong A, Sainsbury P, Craig J. (2007) Consolidated criteria for reporting qualitative research (COREQ): a 32-item checklist for interviews and focus groups. International Journal for Quality in Healthcare: 19:349 – 357

No. Item	Guide questions/description	Reported on Page #
Domain 1: Research team and reflexivity		
1. Inter viewer/facilitator	Which author/s conducted the interview?	7
2. Credentials	What were the researcher's credentials?	1,7
3. Occupation	What was their occupation at the time of the study?	7
4. Gender	Was the researcher male or female?	7
5. Experience and training	What experience or training did the researcher have?	7
6. Relationship with participants established	Was a relationship established prior to study commencement?	8
7. Participant knowledge of the interviewer	What did the participants know about the researcher?	8
8. Interviewer characteristics	What characteristics were reported about the inter viewer/facilitator?	8
Domain 2: study design		
9. Methodological orientation and Theory	What methodological orientation was stated to underpin the study?	6
10. Sampling	How were participants selected?	8
11. Method of approach	How were participants approached?	8
12. Sample size	How many participants were in the study?	8
13. Non-participation	How many people refused to participate or dropped out? Reasons?	8
14. Setting of data collection	Where was the data collected?	6
15. Presence of non-participants	Was anyone else present besides the participants and researchers?	7
16. Description of sample	What are the important characteristics of the sample?	5,6 and Table 1
17. Interview guide	Were questions, prompts, guides provided by the authors?	7
18. Repeat interviews	Were repeat interviews carried out?	9
19. Audio/visual recording	Did the research use audio or visual recording to collect the data?	7
20. Field notes	Were field notes made during and/or	7

For peer review only

	after the inter view?	
21. Duration	What was the duration of the interviews	8
22. Data saturation	Was data saturation discussed?	8
23. Transcripts returned	Were transcripts returned to participants for comment and/or correction?	No
Domain 3: analysis and findings		
24. Number of data coders	How many data coders coded the data?	2
25. Description of the coding tree	Did authors provide a description of the coding tree?	no
26. Derivation of themes	Were themes identified in advance or derived from the data?	8
27. Software	What software, if applicable, was used to manage the data?	No
28. Participant checking	Did participants provide feedback on the findings?	No
29. Quotations presented	Were participant quotations presented to illustrate the themes/findings? Was each quotation identified?	10-16
30. Data and findings consistent	Was there consistency between the data presented and the findings?	10-16
31. Clarity of major themes	Were major themes clearly presented in the findings?	10-16
32. Clarity of minor themes	Is there a description of diverse cases or discussion of minor themes?	10-16

For peer review only
